# Dorsal Root Ganglion Morphometric Changes Under Oxaliplatin Treatment: Longitudinal Assessment by Computed Tomography

**DOI:** 10.1007/s00062-021-01083-5

**Published:** 2021-09-09

**Authors:** Leonidas Apostolidis, Lars Kowalscheck, Tim Frederik Weber, Tim Godel, Martin Bendszus, Hans-Ulrich Kauczor, Dirk Jäger, Heinz-Peter Schlemmer, Philipp Bäumer

**Affiliations:** 1 Department of Medical Oncology, National Center for Tumor Diseases (NCT) Heidelberg, Heidelberg University Hospital, Heidelberg, Germany; 2 Department of Radiology, German Cancer Research Center, Heidelberg, Germany; 3 Department of Diagnostic and Interventional Radiology, Heidelberg University Hospital, Heidelberg, Germany; 4 Department of Neuroradiology, Heidelberg University Hospital, Heidelberg, Germany; 5 Vinzenz-von-Paul Straße 8, 84503 Altötting, Germany

**Keywords:** Computed tomography, Spinal ganglia, CIPN, Oxaliplatin, Chemotherapy, Peripheral neuropathy

## Abstract

**Purpose:**

Magnetic resonance neurography (MRN) can detect dorsal root ganglia (DRG) hypertrophy in patients with oxaliplatin-induced peripheral neuropathy (OXIPN) but is difficult to apply in clinical daily practice. Aims of this study were (i) to assess whether DRG volume is reliably measurable by routine computed tomography (CT) scans, (ii) to measure longitudinal changes in DRG during and after oxaliplatin administration and (iii) to assess correlation between DRG morphometry and individual oxaliplatin dose.

**Methods:**

For comparison of MRN and CT measurements, CT scans of 18 patients from a previous MRN study were analyzed. For longitudinal assessment of DRG size under treatment, 96 patients treated with oxaliplatin between January and December 2014 were enrolled retrospectively. DRG volumetry was performed by analyzing routine CT scans, starting with the last scan before oxaliplatin exposure (*t0*) and up to four consecutive timepoints after initiation of oxaliplatin therapy (*t1–t4*) with the following median and ranges in months: 3.1 (0.4–4.9), 6.2 (5.3–7.8), 10.4 (8.2–11.9), and 18.4 (12.8–49.8).

**Results:**

DRG volume measured in CT showed a moderately strong correlation with MRN (r = 0.51, *p* < 0.001) and a strong correlation between two consecutive CTs (r = 0.77, *p* < 0.001). DRG volume increased after oxaliplatin administration with a maximum at timepoint *t2*. Higher cumulative oxaliplatin exposure was associated with significantly higher absolute DRG volumes (*p* = 0.005). Treatment discontinuation was associated with a nonsignificant trend towards lower relative DRG volume changes (*p* = 0.08).

**Conclusion:**

CT is a reliable method for continuous DRG morphometry; however, since no standardized assessment of OXIPN was performed in this retrospective study, correlations between DRG size, cumulative oxaliplatin dose and clinical symptoms in future prospective studies are needed to establish DRG size as a potential OXIPN biomarker.

**Supplementary Information:**

The online version of this article (10.1007/s00062-021-01083-5) contains supplementary material, which is available to authorized users.

## Introduction

The third-generation platinum compound oxaliplatin (OXA) is used as standard of care in chemotherapy regimens for most gastrointestinal malignancies, including colorectal, gastric and pancreatic cancer [1–3]. OXA has a manageable toxicity profile regarding hematologic, gastrointestinal and renal side effects; however, chronic oxaliplatin-induced peripheral neuropathy (OXIPN) can cause progressive long-term neurological deficits and serious functional difficulties in daily life, severely reducing the quality of life [4, 5]. These adverse effects are dose-limiting, making OXIPN the main cause of dose reductions or discontinuation of OXA-based treatment and compromising therapeutic outcome [6].

OXIPN is dose-dependent and occurs in milder forms (National Cancer Institute common toxicity criteria grade 1–2) in the majority of patients upon completion of a OXA-containing regimen, while severe forms (grade 3–4) are reported in 10–20% of patients [6]. It presents as a distal-symmetric sensory neuropathy with sensory loss, paresthesia, and dysesthesia, and is only incompletely reversible [7].

Accumulation of platinum compounds in the dorsal root ganglia (DRG) of the peripheral nervous system (PNS) is believed to be the main mechanism of OXIPN, causing apoptosis and eventually neuronal atrophy [8]. Directly assessing the extent of damage to the PNS is difficult due to its anatomical dissemination, and electrophysiological methods can only indirectly and functionally investigate the DRG.

A recent study employing magnetic resonance neurography (MRN) showed little changes in peripheral nerves of patients treated with oxaliplatin but demonstrated a significant increase in DRG volume, thus suggesting DRG volume as a potential objective and quantitative correlate for damage to the PNS [9]. The longitudinal course of this oxaliplatin-induced DRG hypertrophy has not yet been examined. If DRG volume were to be used as a biomarker for PNS damage, e.g. in the evaluation of neuroprotective substances counteracting OXIPN, knowledge regarding the time course of DRG hypertrophy would be necessary. Furthermore, it would be important to know whether DRG hypertrophy is reliably observed and quantifiable on an individual basis.

DRG are small structures within the intervertebral foramina of the spine and their measurement depends on sufficient image contrast from the surrounding tissue. The previous study used T2-weighted fat-saturated magnetic resonance imaging (MRI) for DRG morphometry while computed tomography (CT) has not been applied to measure DRG volume so far. Since CT is commonly used for staging of oxaliplatin-treated patients, using imaging data acquired during clinical routine for DRG morphometry would be an easy substitute approach, provided it is a reliable substitute for MRI.

The aims of this retrospective study were therefore the following: firstly to assess whether DRG volume is reliably measurable by CT and corresponds to DRG volume on MRN. Secondly to measure the longitudinal changes in DRG during and after oxaliplatin administration for up to 2 years, and thirdly, to assess the correlation between DRG morphometry with individual oxaliplatin dose.

## Methods

### Patients and Treatment Regimen

This study was approved by the institutional research ethics committee (S-412/2017), and written informed consent from patients was waived due to the retrospective nature of the study.

The study comprised two parts with partially overlapping patient groups. The first part (methodological study) compared MRI and CT measurements of DRG and tested for the most reliable method to quantify DRG volume on CT. The second part (longitudinal study) longitudinally assessed DRG volumes in 96 patients treated with oxaliplatin.

For the methodological study, we used a patient collective from a previous prospective investigation with MRI, with inclusion and exclusion criteria as described in this previous publication [9]. Abdominal CT imaging data from clinical staging CT before and after (median 19 days, defined as timepoints *t*_*x*_ and *t*_*z*_ respectively) the date of the MRI (timepoint *t*_*y*_) were used to compare with each other and with the MRI.

For the longitudinal study, patients were selected by screening the internal pharmaceutical house records of all patients who had received OXA between January and December 2014.

Medical records of these patients were then reviewed for inclusion and exclusion criteria.

Inclusion criteria were defined as follows: age between 18 and 80 years, available abdominal CT imaging data before initiation of OXA treatment (defined as *t0*) and during at least 2 of the following time periods after initiation of treatment: < 5 months (*t1*), 5–< 8 months (*t2*), 8–< 12 months (*t3*), ≥ 12 months (*t4*) and accessibility of comprehensive patient documentation regarding administered treatment.

Exclusion criteria were the following: any history of symptomatic peripheral artery or cerebrovascular disease, alcoholism, end-stage renal disease, or any other disease known to be related to the manifestation of peripheral neuropathy (e.g., autoimmune disease, systemic vasculitis, or infectious diseases) except for chemotherapeutic treatment and diabetes mellitus. Patients with prior treatment containing taxanes (except when applied concomitantly to OXA) were also excluded.

Information on treatment regimens and demographic data as well as discontinuation of therapy due to OXIPN was recorded.

### Imaging

CT examinations were performed on a Philips iCT256 (Philips, Hamburg, Germany) and Siemens Somatom Definition Flash (Siemens, Erlangen, Germany) scanner. Peak voltage was 120 and 100 kV, and pixel sizes were 0.70 and 0.78 mm, respectively. Slice spacing was 1.5 mm, slice thickness was 3 mm.

MRI examinations were performed on a 3 T MR scanner (Magnetom TRIO, Siemens, Erlangen, Germany) with the following pulse sequence: 3D T2-weighted inversion recovery sampling perfection with application-optimized contrasts using different flip angle evolution (SPACE) sequence with 100 axial reformations for imaging of the lumbosacral plexus and spinal nerves: repetition time/echo time (TR/TE) 3000/202 ms, effective TE 68 ms, time of inversion 210 ms, field of view (FOV) 305 × 305 mm^2^, matrix size 320 × 320 × 104, voxel size 1.0 × 1.0 × 1.0 mm^3^.

### Quantitative Measurements

DRG 2D area and 3D volumes were assessed in OsiriX (Pixmeo SARL, Bernex, Switzerland). Reading was predominantly performed by a single observer (LK) who was instructed and supervised by a consultant radiologist (PB) with 9 years of experience in diagnostic radiology. Freak values and a sample of randomly selected measurements were checked and verified by the consultant.

**Fig. 1:**
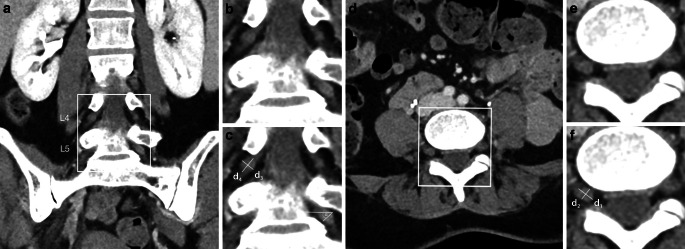
Lumbosacral plexus with dorsal root ganglia. **a** Exemplary frontal plane image from which diameters 3 (*d*_3_), 4 (*d*_4_) and angle (*α*) are measured. **b** Magnification of region of interest in **a**. **c** Application of measurements for *d*_3_, *d*_4_ and *α*. **d** Exemplary axial plane image from which diameters 1 (*d*_1_) and 2 (*d*_2_) are obtained. **e** Magnification of region of interest in **d**. **f** Application of measurements for *d*_1_ and *d*_2_

Slice positions were chosen separately for L4, L5, and S1 DRG and for left and right side each. The largest diameter and its perpendicular in coronal and axial reformations were measured (*d*_1_ to *d*_4_) and the DRG tilt in reference to the axial slice was quantified as angle (*α*) in coronal reformations (Fig. 1). DRG area and volume were then estimated with angle correction by applying the following approximations derived from geometrical equations for area (*A*) of an ellipse and volume (*V*) of an ellipsoid, respectively:

$$A = \pi \left( \frac{\cos \left( \frac{\alpha}{180} \pi \right) d_1 + d_2} {4} \right) \left( \frac{d_3}{2} \right)$$

$$V = \frac{4}{3} \pi \left( \frac{\cos \left( \frac{\alpha }{180} \pi \right) d_1 +d_2}{4} \right) \left( \frac{d_3}{2} \right) \left( \frac{d_4}{2} \right)$$

### Statistical Analysis

All results are expressed as mean ± standard deviation. In group comparisons, Student’s t‑test was used with the significance level set to *p* < 0.05.

Relative values for each individual DRG area and volume were obtained by dividing each value for every level at a defined time point *t1–t4* to its own individual baseline *t0*. Resulting quotients were logarithmized to reduce skewness.

Absolute values for DRG area and volume were averaged over all individuals for each level at a defined time point (*t0–t4* as defined in the inclusion criteria).

Average individual DRG values for each patient were calculated by averaging L4, L5, and S1 for left and right side.

Mean values were compared between the observed time points during and/or after treatment.

Group comparisons were drawn to assess predictability of treatment discontinuation at a certain cumulative dose or DRG size threshold by performing logistic regression analysis. Mean values were compared between the patient group with a cumulative OXA dose < 900 mg/m^2^ versus the patient group with higher exposure.

Correlation analysis was performed to assess CT to CT and MRI to CT reliability and cumulative dose to DRG size relationship by calculating Pearson’s correlation coefficient.

Statistical analysis was performed in Microsoft Excel version 15.34 (Microsoft, Redmond, WA, USA), IBM SPSS Statistics 22 (IBM, Armonk, NY, USA) and SigmaPlot 13.0 (Systat Software Inc., Erkrath, Germany).

## Results

### Methodological Study

#### Reliability of Methods

For a first test of the reliability of CT-based DRG measurements, 18 patients (6 female, 12 male, mean age 58.3 ± 9.1 years) who underwent both CT and MRI scans at comparable time points were assessed. The average cumulative OXA dose applied was 689.3 ± 278.2 mg/m^2^ (range 255–1275 mg/m^2^).

**Fig. 2:**
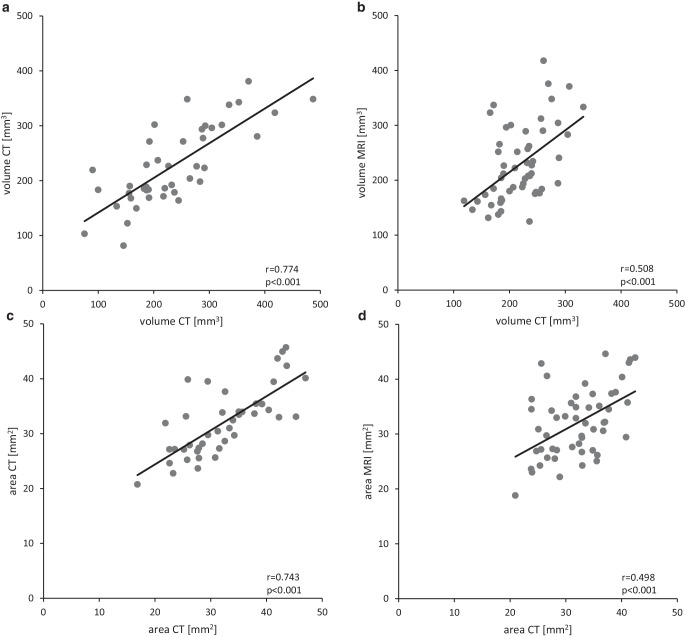
Comparison between CT to CT and CT to MRI correlation. Correlation of volume measured by CT at *t*_*x*_ (x axis) with CT at *t*_*z*_ (y axis) (**a**). Correlation of volume measured by CT mean with MRI at *t*_*y*_ (**b**). Correlation of area measured by CT at *t*_*x*_ (x axis) with CT at *t*_*z*_ (y axis) (**c**). Correlation of area measured by CT mean with MRI at *t*_*y*_ (**d**)

To determine the most suitable size parameter for DRG assessment and reliability of consecutive measurements, both volume and area were compared between MRI and CTs before and after the MRI (Fig. 2).

The DRG size measured in CT (t_x_) showed a moderate correlation with MRI (t_y_) for area (r = 0.50, *p* < 0.001) as well as for volume (r = 0.51, *p* < 0.001).

Furthermore, DRG area measured in two consecutive CTs (t_x_, t_z_) showed good reliability of values (r = 0.74, *p* < 0.001), but DRG volume resulted in a slightly stronger correlation (r = 0.77, *p* < 0.001).

We concluded that DRG size measurements on CT clinical staging datasets are reliable, and that volume was a slightly more reliable measure than area. Any further measurement in the following study was therefore based on volume.

### Longitudinal Study

After establishing methods and confirming their reliability, the second, longitudinal study was performed. A total of 362 patients with OXA treatment were identified. Of those, sufficient imaging data at the prespecified time points were available for 141 patients. Sufficient medical documentation could be retrieved for 132 patients. After application of the predefined exclusion criteria, 96 patients (38 female, 58 male, 60.8 ± 9.7 years) were included with an average cumulative OXA dose of 750 ± 373 mg/m^2^ (range 85–2295 mg/m^2^) (Table 1). The applied treatment protocols included FOLFOX [3], FOLFIRINOX [2], FLO [10], FLOT [1], OFF [11] and XELOX [12]. During the observational period (23.3 ± 8.8 months, range 14.5–51.5 months) 5 time points were evaluated (Table 2) and 32 patients discontinued OXA treatment because of OXIPN symptoms of polyneuropathy before being administered final planned doses.

**Table 1:** Patient characteristics of the longitudinal study

Characteristic			Number (*n* = 96)	%
Sex	Male	–	58	60.4
	Female	–	38	39.6
Age (years)	Median	61	–	–
	Range	44–80	–	–
Primary tumor	Colorectal cancer	–	40	41.7
	Esophageal/gastric cancer	–	21	21.9
	Pancreatic cancer	–	24	25.0
	Biliary tract cancer	–	7	7.3
	CUP	–	1	1.0
	NET	–	2	2.1
	Liver stem cell	–	1	1.0
Treatment protocol	FOLFOX	–	50	50.2
	FOLFIRINOX	–	20	20.1
	FLO	–	13	13.5
	FLOT	–	11	11.5
	OFF	–	8	8.3
	XELOX	–	1	1.0
Cumulative OXA dose (mg/m^2^)	Mean	750	–	–
	Range	85–2295	–	–
	< 900	–	63	65.6
	> 900	–	33	34.4
OXA discontinuation	Yes	–	32	33.3

*CUP* cancer of unknown primary, *NET* neuroendocrine tumor, *OXA* oxaliplatin

**Table 2:** Imaging timepoint characteristics

	*t0*	*t1*	*t2*	*t3*	*t4*
Time after first OXA administration (months)					
Median	–	3.1	6.2	10.4	18.4
Range	–	0.3–4.9	5.3–7.8	8.2–11.9	12.8–49.8
Patients with imaging	96	84	80	68	49
Patients with imaging still receiving OXA	0	68	39	19	10

*OXA* oxaliplatin

#### Longitudinal Volumetry

**Fig. 3:**
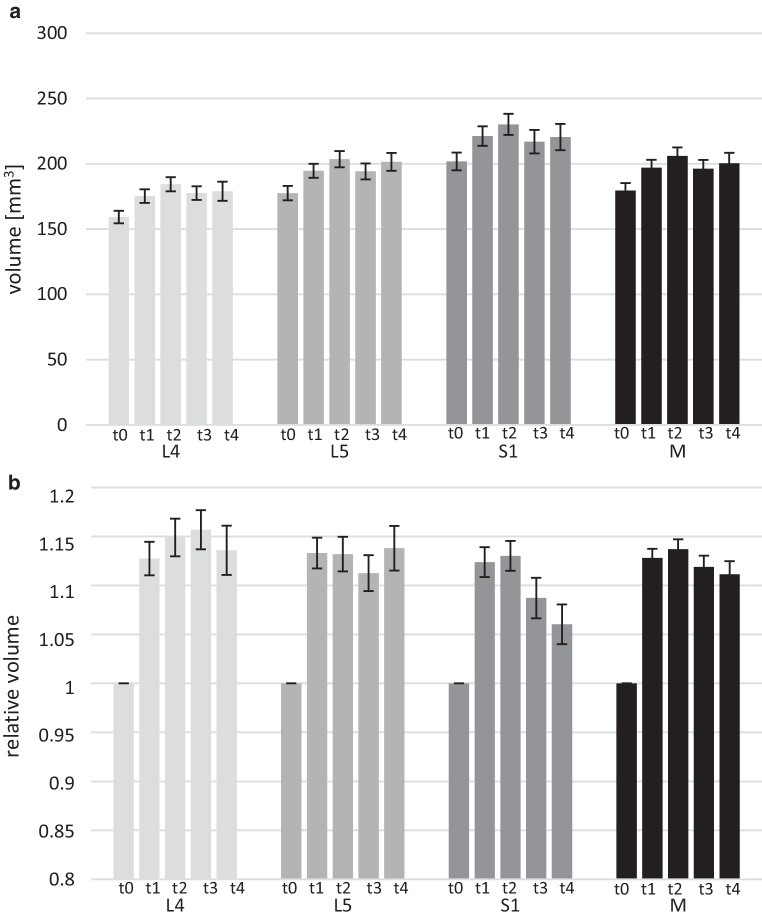
Longitudinal volumetry. The upper chart shows mean absolute volumes for every time point (*t0–t4*) at every DRG level (L4, L5, S1), and averaged over all DRG levels (M) (**a**). The lower chart shows mean relative volumes for every time point (*t0–t4*) at every DRG level (L4, L5, S1), and averaged over all DRG levels (M) with baseline (*t0*) set to 1 (**b**)

Significant DRG hypertrophy after OXA administration was noted (Fig. 3). Mean absolute DRG volume before treatment application (*t0*) was 179.4 ± 58.5 mm^3^ and then rose to 196.9 ± 57.4 mm^3^ (*p* < 0.001) at *t1*, a maximum of 206.1 ± 60.4 mm^3^ (*p* < 0.001) at *t2*, 196.2 ± 57.0 mm^3^ (*p* = 0.002) at *t3*, and 200.3 ± 58.0 mm^3^ (*p* < 0.001) at *t4*.

Relative DRG volume likewise exhibited this increase from baseline (*t0* by definition at 1.0) to 1.13 ± 0.14 (*p* < 0.001) at *t1*, a maximum of 1.14 ± 0.15 (*p* < 0.001) at *t2*, 1.12 ± 0.16 (*p* < 0.001) at *t3*, and 1.11 ± 0.15 (*p* < 0.001) at *t4*.

This pattern of a steep increase in volume within the first 7 months of treatment and a subsequent minor decline from a maximum was consistently noted for both absolute and relative DRG values.

#### Correlation to dose

Patients with higher cumulative exposure to OXA (> 900 mg/m^2^) had significantly higher absolute DRG volume values with 217.24 ± 44.0 mm^3^ versus 189.78 ± 43.1 mm^3^ (*p* = 0.005). This difference was not significant when comparing relative DRG volumes (1.12 ± 0.09 vs. 1.13 ± 0.10, *p* = 0.66).

No significant absolute DRG size differences were observed between those patients with and those without discontinuation of OXA treatment (197.82 ± 38.93 mm^3^ vs. 200.28 ± 48.48 mm^3^, *p* = 0.805). Patients who discontinued treatment had lower relative DRG volumes, although this trend did not reach statistical significance in two-sided tests (1.10 ± 0.10 vs. 1.13 ± 0.09 vs., *p* = 0.078) (Table 3). Cumulative dose was not responsible for this difference because patients with discontinuation of treatment were on average applied higher doses (807.10 ± 269.58 mg/m^2^) than those without (718.33 ± 415.54 mg/m^2^).

**Table 3:** Comparison of volume with and without OXIPN-associated therapy discontinuation

	OXA discontinued	OXA not discontinued	*p* value
Max volume (mm^3^)	276.88 ± 61.78	284.46 ± 70.52	0.609
Mean volume (mm^3^)	197.82 ± 38.93	200.28 ± 48.48	0.805
Max relative volume	1.57 ± 0.21	1.59 ± 0.15	0.712
Mean relative volume	1.10 ± 0.10	1.13 ± 0.09	0.078

*OXA* oxaliplatin, *OXIPN* oxaliplatin-induced peripheral neuropathy

**Fig. 4:**
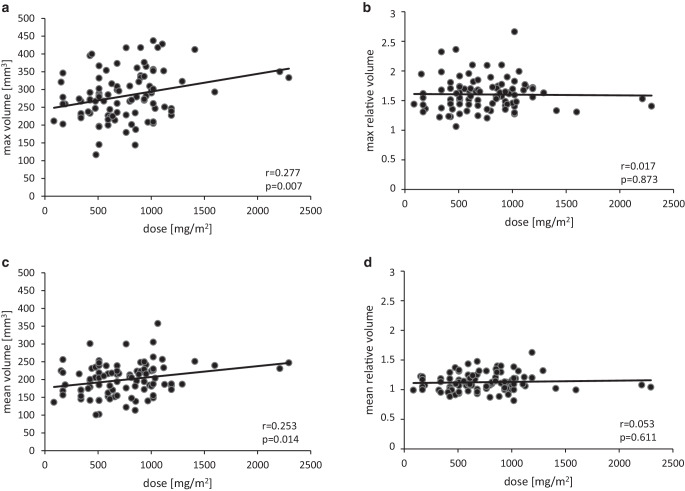
Volume to dose correlation. **a** Correlation of cumulative dose administered and maximum absolute volume. **b** Correlation of cumulative dose administered and maximum relative volume. **c** Correlation of cumulative dose administered and mean absolute volume. **d** Correlation of cumulative dose administered and mean relative volume

Cumulative OXA dose was not strongly associated with mean or maximum DRG volume. While absolute DRG volumes had a significant but not very strong correlation with dose, (r_mean_ = 0.253, *p* = 0.014; r_max_ = 0.277, *p* = 0.007) no meaningful correlation of relative volume increase with dose was found (r_mean_ = 0.053, *p* = 0.611; r_max_ = 0.017, *p* = 0.873) (Fig. 4).

Furthermore, no significant relationship between mean absolute or relative volume and discontinuation of treatment controlling for the variables sex, age, and dose was found.

## Discussion

This study investigated DRG morphometry in patients under OXA treatment. It establishes computed tomography as a reliable modality to assess DRG volume and characterizes the course of DRG volume under and after treatment for almost 2 years.

In-vivo measurement of DRG volume has been under investigation in recent studies in different diseases such as Fabry’s disease, neurofibromatosis 2, or schwannomatosis [13, 14]. DRG size in patients under neurotoxic treatment had been investigated in one prior study by using MRN [9]. While this modality is without doubt the best and most accurate method to investigate the peripheral nervous system by imaging, it is very ambitious to recruit a large number of patients for consecutive MRN measurements for sole scientific purposes. This is further complicated by their relatively high morbidity due to the underlying disease and ongoing treatment as in the case of OXA.

For these reasons, we sought to find a different approach to undertake DRG morphometry in a large number of patients. Using CT data from clinical staging, this study assessed the reliability of DRG morphometry by standard CT and found that it is sufficiently reliable to undertake further analyses using the established methods. Notably, due to the angular positioning of DRGs near the intervertebral foramina, angle correction is useful in the formula to obtain more exact calculations of DRG volume. Volume was found to be slightly more accurate than cross-sectional area, but both markers are feasible.

After establishing the methodology, a large number of patients were identified and included to characterize the course of DRG volume changes during and after OXA administration. In the previous MRI study, significant DRG increase was described for patients as opposed to controls at a single time point [9]. This stood in contrast to both experimental animal and to postmortem patient publications, which described DRG hypotrophy in platinum treated animals and patients [15–20]. The present study confirms hypertrophy by in vivo imaging. The hypotrophy described in the earlier publications is therefore likely due to different, non-platinum associated effects such as postmortem involution or possibly loss of blood volume, since DRGs are especially strongly perfused tissue [21], with postmortem loss of blood tension resulting in loss of DRG volume. Additionally, other publications investigating DRG size in disease by MRN mostly reported an increase in size [13, 14].

The present study further advances the findings of the previous MRN study by describing the longitudinal course of DRG volume. We describe an initial increase in size within the first 7 months with a subsequent minor decline of volume.

The longitudinal design of the analysis further allowed to normalize intraindividual DRG values to a baseline before OXA treatment. Using this approach, we found an average increase of 14% in volume after 7 months of treatment. Although this increase is statistically significant, the increment is quite small and difficult to discern by visual impression alone, making the volumetry necessary to assess those changes. The previous MRI study found a stronger increase (37%) in patients over controls, and it is possible that CT underestimated this increase in size.

As a third aim of the study, we set out to assess potential correlations between DRG volume increase with individual cumulative OXA dose and with OXIPN-associated discontinuation of treatment. The interpretation of the results is complex because absolute and relative values of DRG size yielded different associations with dose and discontinuation. While significant positive correlations were found between absolute size and cumulative dose, relative values showed no such association. Given these findings, no satisfactory conclusions can be drawn at present, but this issue might be worthwhile in future studies with more detailed and standardized neurological test results.

Surprisingly, patients who discontinued treatment as a measure of higher subjective damage to the PNS, had lower relative increase in size. If this (barely non-significant) trend was true, it might indicate that DRG size increase is the measurable correlate of a normal response to a toxic agent and failure to do so results in more symptoms.

The OXA dose is administered according to body surface area [1–3]. Given the discrepancies between the correlations of absolute and relative values, it seems also conceivable that body surface area as an indicator of OXA dosing is not entirely adequate regarding PNS side effects. While this approach to chemotherapy dosing has been a major advance and is considered standard of care [22], multiple studies have questioned the use of body surface area for optimal dosing in a number of cytotoxic agents [23–25]. Besides cumulative dose, several other clinical [26] and molecular predictors [27] of OXIPN are under investigation. In the future, for a more personalized multidimensional dosing approach, DRG volume may be considered an additional helpful indicator in dosage of OXA or other chemotherapeutic agents with damaging effects to DRG resulting in sensory polyneuropathy.

There are several limitations to the study, all of which can be attributed to its retrospective nature. First and mainly, a standardized neurological assessment of the PNS was not obtained. Therefore, it was not possible to reliably assess intensity or grade of OXIPN in the examined patients. Since sensory polyneuropathy was described for nearly all patients, we could therefore correlate the results only to the binary result of OXIPN-associated treatment discontinuation versus administration of full planned dose. Second, CT slices available for analyses were somewhat thicker than would have been chosen for this purpose in a prospective manner. This might help to explain the stronger effect of DRG size increase in MRN versus this study and third, the time points chosen for analysis included a range during which the clinical CT was performed, such that actual time points varied somewhat among patients. This was especially true for the last timepoint t4, having been performed after a median of 18.4 months but spanning a range of more than 3 years. And finally, the data were retrieved several years ago; however, neither the importance of oxaliplatin nor the very limited treatment options for OXIPN management have relevantly changed in this time period.

In conclusion, we could establish CT as a reliable method for DRG morphometry. During OXA therapy, DRG increase in size for the first 7 months and then decrease in size but remain enlarged; however, since no standardized assessment of OXIPN was performed in this retrospective study, the correlation between intraindividually normalized DRG size and subjective symptoms will have to be further investigated in future prospective studies to assess if the use of DRG size might be an objective, quantitative biomarker for damage to the sensory PNS.

## Supplementary Information

**Exemplary correlation of each individual DRG volume measurement on level S1 comparing right to left side. **All volumes are in mm^3^. Right side is depicted on the x axis, left side on the y axis
