# Supplementary material for: Dorsal Root Ganglion Morphometric Changes Under Oxaliplatin Treatment: Longitudinal Assessment by Computed Tomography
Source: Clin Neuroradiol. 2021 Sep 9;32(2):547–56. doi: 10.1007/s00062-021-01083-5 (PMC9187544; doi:10.1007/s00062-021-01083-5)
Supplement: Supplementary file 1 — Exemplary correlation of each individual DRG volume measurement on level S1 comparing right to left side. All volumes are in mm3. Right side is depicted on the x axis, left side on the y axis [file 62_2021_1083_MOESM1_ESM.docx]

**Supplemental figure 1. Exemplary correlation of each individual DRG volume measurement on level S1 comparing right to left side.**

All volumes are in mm³. Right side is depicted on the y axis, left side on the y axis.
